# Ce:LYSO, from scintillator to solid-state lighting as a blue luminescent concentrator

**DOI:** 10.1038/s41598-023-32689-z

**Published:** 2023-05-03

**Authors:** Lisa Lopez, Pierre Pichon, Pascal Loiseau, Bruno Viana, Rachid Mahiou, Frederic Druon, Patrick Georges, François Balembois

**Affiliations:** 1 Université Paris-Saclay, Institut d’Optique Graduate School, CNRS, Laboratoire Charles Fabry, 91127 Palaiseau, France; 2 Université PSL, Chimie ParisTech, CNRS, Institut de Recherche de Chimie Paris, 75005 Paris, France; 3 Université Clermont Auvergne, CNRS, Clermont Auvergne INP, ICCF, 63000 Clermont–Ferrand, France

**Keywords:** Lasers, LEDs and light sources, Materials for optics, Lasers, LEDs and light sources, Inorganic LEDs

## Abstract

Cerium-doped lutetium-yttrium oxyorthosilicate (Ce:LYSO) is a well-known single crystal scintillator used in medical imaging and security scanners. Recent development of high power UV LED, matching its absorption band, questions the possibility to use Ce:LYSO in a new way: as LED-pumped solid-state light source. Since Ce:LYSO is available in large size crystals, we investigate its potential as a luminescent concentrator. This paper reports an extensive study of the performance in close relation to the spectroscopic properties of this crystal. It gives the reasons why the Ce:LYSO crystal tested in this study is less efficient than Ce:YAG for luminescent concentration: limited quantum efficiency and high losses coming from self-absorption and from excited-state absorption are playing key roles. However, we demonstrate that a Ce:LYSO luminescent concentrator is an innovative source for solid-state lighting. Pumped by a peak power of 3400 W in quasi-continuous wave regime (40 µs, 10 Hz), a rectangular (1 × 22 × 105 mm^3^) Ce:LYSO crystal delivers a broadband spectrum (60 nm FWHM) centered at 430 nm. At full output aperture (20 × 1 mm^2^), it emits a peak power of 116 W. On a squared output surface (1 × 1 mm^2^) it emits 16 W corresponding to a brightness of 509 W cm^–2^ sr^–1^. This combination of spectrum power and brightness is higher than blue LEDs and opens perspectives for Ce:LYSO in the field of illumination namely for imaging.

## Introduction

Among the materials for solid-state lighting, Ce:YAG is certainly the crystal with the longest history. Its spectroscopy has been extensively studied in the 1970s^1–3^. It started to be used as scintillator in the 1990s^4^ and it is broadly used for beta radiation or X-ray counting, electron or X-ray imaging screens. The first use as solid-state lighting is reported in 1997^5^, with a wavelength conversion by absorption of a blue GaN LED and an emission in the yellow-orange. Next Ce:YAG has been used as a light conversion phosphor in multiple configurations: powders^6^, ceramics^7^, single crystals pumped by blue lasers diodes^8^, and more recently LED-pumped Ce:YAG luminescent concentrators^9–12^.

**Figure 1:**
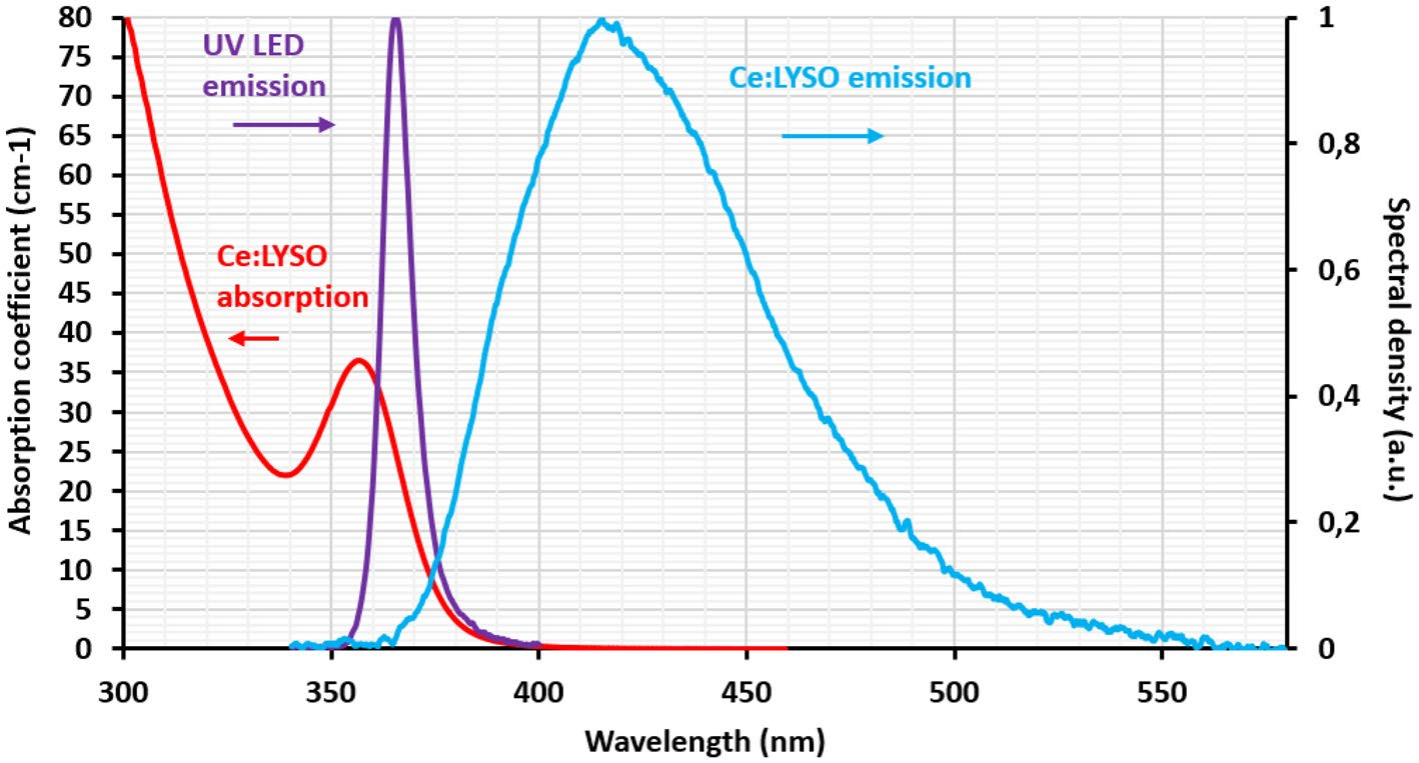
b-cut Ce:LYSO absorption and emission spectra compared to 365 nm UV LED emission spectrum (Nichia , NVCUQ096A-U365). None of the light sources are polarized.

During the last 30 years, many materials were selected and developed for scintillator applications^13^. As this field requires large size crystals with good optical quality, it is interesting to investigate if any other crystal dedicated to scintillation have a potential for solid-state lighting and for new luminescent concentrators. Among them, Ce:LYSO (Ce:Lu_2(1−x)_Y_2x_SiO_5_) is known to be an excellent scintillator for positron emission tomography (PET-scan), calorimetry in high energy physics, or security scanners. Investigations on this crystal started 20 years ago^14–16^. Its crystal growth process is now well mastered. Ce:LYSO is commercially available in large dimensions (up to 100 mm) with excellent optical quality for scintillation applications. Ce:LYSO absorption band around 350 nm matches quite well the emission spectrum of high power UV LEDs that have been recently developed for optical adhesive curing, sterilisation or bio/medical treatments. Moreover, Ce:LYSO emits a broadband spectrum that complete the Ce:YAG spectrum in the bluer range (Fig. 1). Therefore, Ce:LYSO appears to be a good candidate for a LED-pumped luminescent concentrator emitting in the blue.

The purpose of this work is to investigate its potential through an extensive experimental study supported by an analytical model. Firstly, we briefly investigate the spectroscopy of Ce:LYSO under optical pumping in the UV. Then, we present the experimental setup of a LED-pumped Ce:LYSO luminescent concentrator and the performance in terms of peak power and brightness. Afterward, we confront the experimental results with an analytical model showing the origin of the limitations in Ce:LYSO performance. Finally, we compare this new light source to other blue sources and conclude about the potential of LED-pumped Ce:LYSO luminescent concentrators.

## Spectroscopic investigations

To our knowledge, this is the first time that a Ce:LYSO is optically pumped in the UV. Samples used in this work are large single crystals from Crytur.cz company. Emission and absorption spectroscopy are performed using a small sample of 10 × 2 × 1 mm^3^. Absorption measurements are carried out with a UV/Vis/NIR spectrophotometer (Varian Cary 6000). The spectra are reported in Fig. 1. The crystal orientation has been identified with Laue X-ray diffraction method. Absorption and emission are measured for polarizations parallel to the two different dielectric axes perpendicular to b monoclinic axis. No significant differences have been observed between the different polarizations, as reported previously^17^. From the absorption measurements, we deduced that the Ce^3+^ concentration is 0.05% in our samples.

Quantum yields efficiencies were assessed using C9920-02G PL-QY measurement system from Hamamatsu consisting in a 150 W monochromatized Xe lamp, an integrating sphere (Spectralon coating, ∅ = 8.4 cm) and a high sensibility CCD camera. We experimentally obtained a quantum efficiency of ${\eta }_{r}=$ 0.51 for a pump wavelength at 365 nm, corresponding to the operating central wavelength of the LED that will be used for pumping a Ce:LYSO luminescent concentrator. With the same experimental setup and for a sake of comparison, we also measure a quantum efficiency for a commercial Ce:YAG (PhosphorTech) pumped at 460 nm (its excitation wavelength). This value for Ce:YAG is significantly higher with ${\eta }_{r}=$ 0.95.

Two hypotheses can be put forward to understand this relatively low quantum efficiency. A first possible explanation comes from the position of the 5d bands within the Ce:LYSO bandgap. The lowest 5d level (namely 5d^1^ level) of Ce^3+^ is located inside the bandgap, ensuring a good emission, but the second and third lowest 5d levels are located within the conduction band. Therefore a low quantum efficiency could be related to a quenching process in the Ce:LYSO host in relation with the thermally stimulated ionization process from the lowest 5d^1^ level to the conduction band. This process has been widely reported in the garnets for instance comparing Ce:YAG and Ce:YAGG (Ce:Y_3_Al_2_Ga_3_O_12_)^18^. Similar situation has also been observed in various oxides compounds^19^. A second explanation comes from the absorption spectrum of our Ce:LYSO samples as presented in Fig. 1. Indeed, the rising absorption front below 320 nm can be related to Ce^4+^ species. Tetravalent cerium is actually required to improve scintillation light yield, as reported in references^20,21^, and its presence is typically promoted by the crystal growth atmosphere containing a little amount of dioxygen. However, Ce^4+^ species can absorb the pump light in the place of Ce^3+^ and hence reduce the emission in the blue. To corroborate the effect of the tetravalent cerium on the quantum efficiency, a thermal annealing was performed under reducing atmosphere on a small sample, and this indeed increase the quantum yield. However, we observed that the optical quality of the sample was affected. Thermal annealed large pieces of Ce:LYSO would certainly require more investigations that are out of the purpose of this work which presents the use of commercially available Ce:LYSO crystals optimized for scintillators.

LYSO structure presents also two distinct crystallographic sites for Ce^3+^ions, one in coordination 6 and the other in coordination 7 with a more distorted polyhedron. The two resulting Ce^3+^ centers, usually referred as Ce1 and Ce2, have different optical properties and hence different quantum yields. Ce1 has the highest quantum yield, measured at 78% at T = 14 K^22^, with main emission peaks at 393 and 427 nm^23^, whereas Ce2 has weaker quantum yield although it is the most abundant^24^ with main emission peak around 460 nm. As Ce1 and Ce2 centers notably present excitation bands at 356 and 376 nm respectively^23^, both are excited when performing room temperature excitation at 365 nm, so that the resulting mean quantum yield is an average between the two centers contributions.

**Figure 2:**
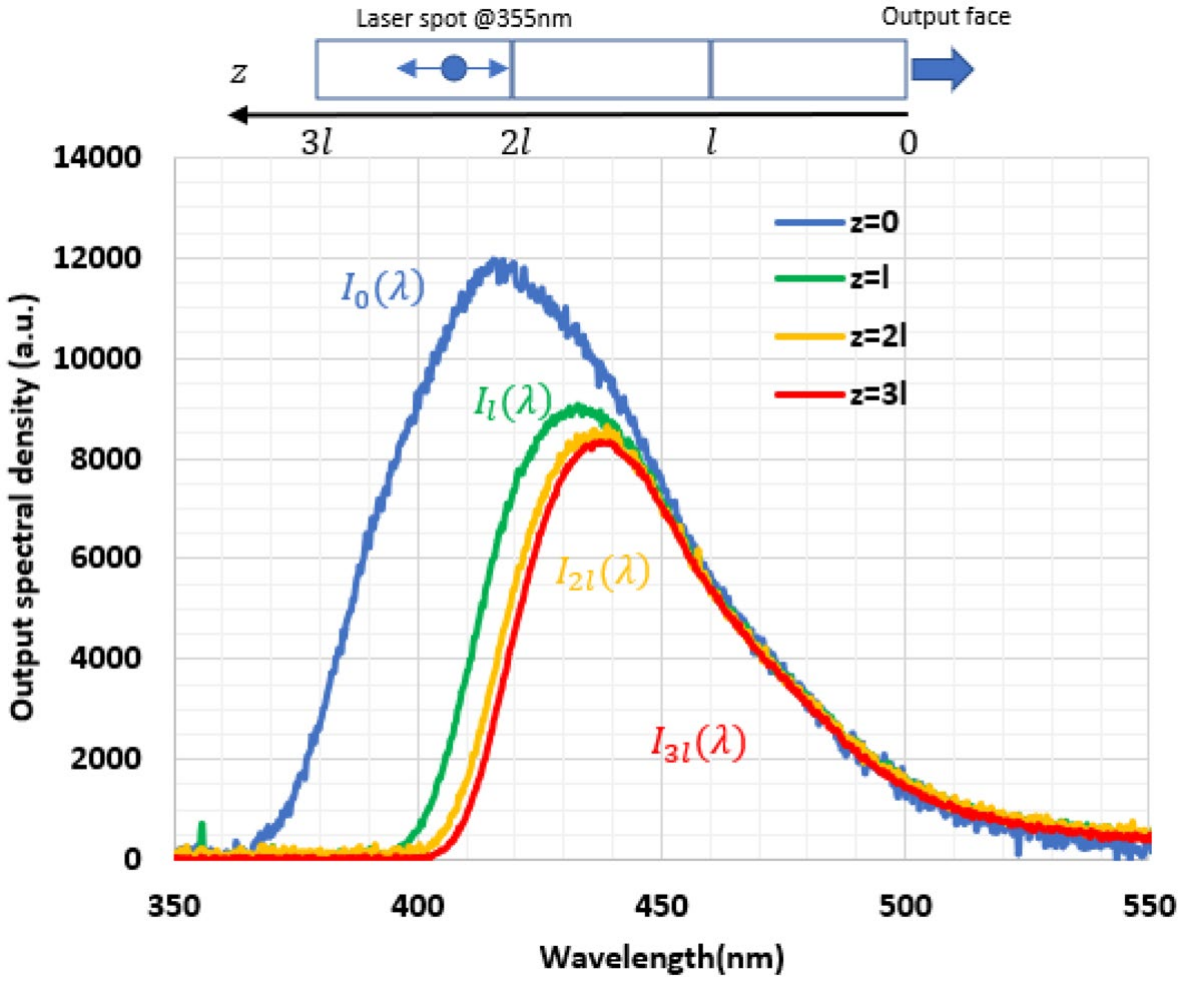
Ce:LYSO emission spectra recorded at the output face and pumped by a frequency tripled Nd:YAG laser emitting at 355 nm. l = 105 mm. The sample dimensions are 1 × 22 × 315 mm^3^ with l = 105 mm. Spectra are normalized for wavelengths larger than 450 nm.

As the overlap between the absorption spectrum and the emission spectrum is significant (Fig. 1), we investigate the emission spectrum versus different propagation lengths in the Ce:LYSO. For this purpose, we use a frequency tripled Nd:YAG as pump source (emission at 355 nm) and move its 5 mm diameter pump spot along our 315-mm-long Ce:LYSO sample. This sample is composed of 3 parallelepiped Ce:LYSO single all-faces-polished crystals (each of them having dimensions of 1 × 22 × 105 mm^3^). The parallelepipeds are bonded together with a UV-curing optical adhesive (refractive index of n = 1.56) to minimize reflections at the interface between bonded crystals. Ce:LYSO crystal absorbs the UV light at 355 nm and emits a blue light localized in the pump spot. A part of this converted light propagates in the sample by total internal reflections and reaches the output surface where the emitting spectrum is collected with an integrating sphere connected to a spectrometer. The different output spectra shown in Fig. 2 are normalized assuming that self-absorption is negligible for wavelengths larger than 450 nm and that the spectra are then similar in the reddest regions. Figure 2 shows a significant evolution of the emission spectrum versus the propagation length inside the crystal. This can be related to self-absorption losses for light guided inside the crystal by total internal reflections (TIR). Indeed, after each self-absorption-re-emission process, the light is redistributed in all directions and part of it can again escape through the faces of the crystal. The knowledge of these losses is thus crucial for luminescent concentrators.

In order to evaluate these losses, one can consider the propagation over a distance $l$ as a first example. The spectral densities to be compared are ${I}_{0}(\lambda )$ and ${I}_{l}(\lambda )$ (Fig. 2), measured when the pump spot is close to the output surface (at z = 0) and at a distance z = $l$, respectively. The photons coming from the pump spot at $z=l$ propagate in the crystal towards the output face by TIR in directions included in a cone (called the output escape cone) with an apex angle ${\theta }_{TIR}={sin}^{-1}\left(\frac{1}{n}\right)$, $n$ being the refractive index of the crystal. In^22^ we have shown that the average propagation distance is $d=Kl$ with

1 $$K=\frac{-ln(\mathrm{cos}\left.{\uptheta }_{TIR}\right)}{\left(1-\mathrm{cos}{\uptheta }_{TIR}\right)}$$

For each wavelength, one can deduce a loss coefficient caused by self-absorption given by:

2 $$\alpha \left(\lambda \right)=-\frac{1}{Kl}\mathrm{ln}\left(\frac{{I}_{l}\left(\lambda \right)}{{I}_{0}(\lambda )}\right)$$

Average self-absorption losses coefficient ${\alpha }_{l}$ can be found by integrating all over the output spectrum:

3 $${\alpha }_{l}=\frac{{\int }_{390\mathrm{nm}}^{550\mathrm{nm}}\alpha (\lambda ){I}_{l}\left(\lambda \right)d\lambda }{{\int }_{390\mathrm{nm}}^{550\mathrm{nm}}{I}_{l}\left(\lambda \right)d\lambda }$$

With the data recorded on the experiment described on Fig. 2, we found ${\alpha }_{l}$ = 1.7 × 10^–2^ cm^−1^.

For comparison, we propagate a HeNe laser (at 633 nm) in the Ce:LYSO crystal, along 105 mm (without reflexions on faces). We found a loss coefficient of α_0_ = 10^–3^ cm^−1^ showing the excellent optical quality of our sample. This means that self-absorption losses are much more significant than losses due to crystal imperfections, the assumption of normalization at long wavelengths is thus validated.

## LED-pumped Ce:LYSO luminescent concentrator: experimental setup

**Figure 3:**
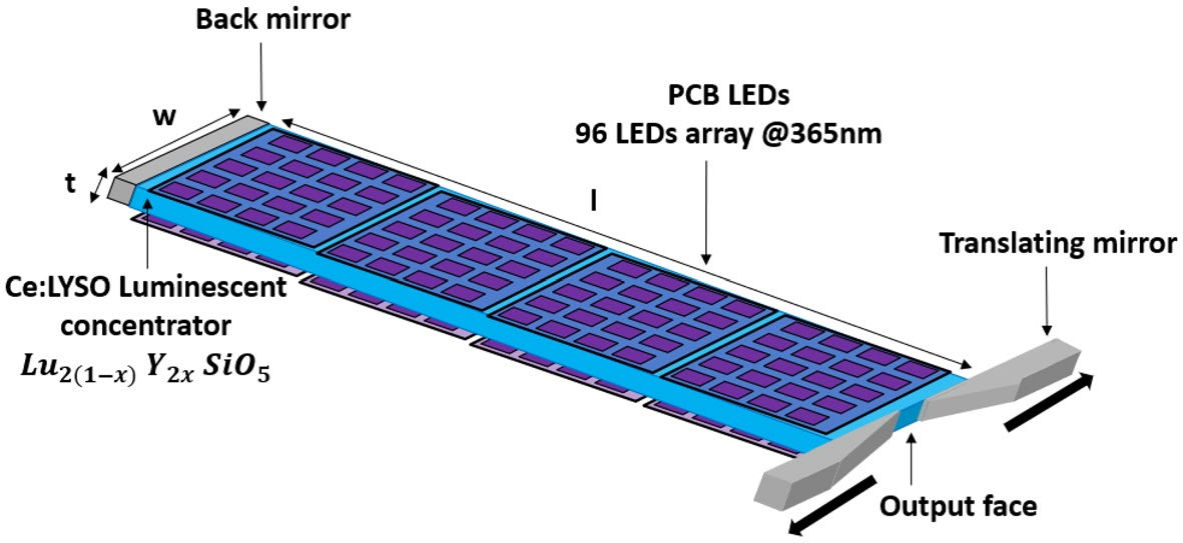
Experimental setup of the LED pumped Ce:LYSO luminescent concentrator. The b-cut Ce:LYSO dimensions are $t$ = 1 mm, $w$ = 22 mm and $l$ = 105 mm. Beveled translating mirrors on the output face are used to adjust the output surface and the brightness.

The experimental setup of the LED-pumped luminescent concentrator is presented in Fig. 3. The Ce:LYSO concentrator is cut as a parallelepiped, whose length, width, and thickness are $l$ = 105 mm, $w$ = 22 mm and $t$= 1 mm, respectively. The six faces of the Ce:LYSO are optically polished with a scratch/dig of 60/40. The parallelism between the two large faces "$w.l$" is measured to be 3.4 λ (peak to valley) at 633 nm, by interferometry.

The LC is pumped on both sides, across its two largest ($w.l$) surfaces by 768 UV LEDs arranged in 8 arrays (Nichia, NVCUQ096A-U365), brazed on ceramic printed circuit boards (PCB). The PCBs are mounted on aluminum blocks with water cooling for temperature control. Each array is composed of 96 LEDs divided in 12 lines of 8 LEDs. The 8 arrays are represented on Fig. 3. They are close to the large faces of the Ce:LYSO (typically at 1 mm). Each PCB can provide an optical power of 123 W in continuous wave (CW) for a nominal drive current of 8 A corresponding to a voltage of 46.3 V. The LED emission spectrum is centered at 365.5 nm and has spectral width of 10 nm at full width at half maximum (Fig. 1). In order to increase the output peak power, the LEDs are activated in quasi continuous wave (QCW), during 40 μs at 10 Hz. At this very low duty cycle (0.04%), it is possible to increase the drive current up to 30 A, corresponding to a peak power of 426 W per PCB. The total peak power available is then 3405 W.

The spectrum of the LEDs at maximum current is shown on Fig. 1. The spectral overlap of UV LEDs emission with Ce:LYSO concentrator absorption band gives an average absorption coefficient of ${\alpha }_{p} =$ 17.7 cm^−1^ (see Table 1).

**Table 1:** Physical parameters of Ce:LYSO as luminescent concentrator compared with Ce:YAG. For the Ce:YAG, data come from^12^ excepted when it is mentioned in the table.

Parameter description	Ce:YAG (0.11% doping)	Ce:LYSO (this work) (0.05% doping)	Comments for Ce:LYSO
Refractive index	n = 1.82	n = 1.83	
Pump wavelength (peak)	λ_p_ = 440 nm	λ_p_ = 365 nm	Mean wavelength of the LED
Absorption coefficient	α_p_ = 18 cm^−1^	α_p_ = 17.7 cm^−1^	Averaged over the LED spectrum
Emitted wavelength	λ_em_ = 550 nm	λ_em_ = 450 nm	Mean wavelength of the emitted spectrum
Liftetime of the emitting level	τ = 70 ns^4^	τ = 41 ns	From measurement, in agreement to^14^
Luminescence quantum efficiency	${\eta }_{r}=0.95$	${\eta }_{r}=0.51$	
Diffusion loss coefficient	$7 \times {10}^{-4}{\mathrm{ cm}}^{-1}$^25^	${10}^{-3}{\mathrm{ cm}}^{-1}$	Measured at 633 nm
Self-absorption loss coefficient	$1.6\times {10}^{-3}{\mathrm{ cm}}^{-1}$ ^25^	$4\times {10}^{-3}{\mathrm{ cm}}^{-1}$	Deduced from measurements for propagation distances larger than z = 20 cm, correspond to ${\alpha }_{3l}$
Excited state absorption cross section	$1.2 \times {10}^{-17} {\mathrm{cm}}^{2}$	$2.5\times {10}^{-17} {\mathrm{cm}}^{2}$	Fit parameter adjusted for ${P}_{3D}$ at a pump peak power of 3405 W

In order to preserve total internal reflections in the concentrator, Ce:LYSO is maintained by 4 small pins. This means that the Ce:LYSO is quasi exclusively radiatively cooled. With a duty cycle of 0.04%, the average pump power reaches a maximum of 1 W. Assuming that a maximum of 50% of the power is converted into heat (corresponding to the quantum efficiency), we calculated a temperature increase of 7 °C. The quantum efficiency of the Ce:LYSO is temperature dependent^15^ but performance are only slightly affected by this small temperature increase.

The output face of the system is one of the small faces (surface $S=w.t$). In order to increase the output power and the brigthness, mirrors (with the E02 coating from Thorlabs: HR 400–750 nm) can be positioned close to the $w.t$ faces. A so-called "back" mirror (Fig. 3) put on the opposite face to the output face then allows to recycle the rays emitted in the backward direction compared to the output face. This is a classical configuration already reported in the literature^9–11^. In the following, this configuration refers as a "2D" configuration because the light emerging on the output face has been confined in the two lateral directions of space by total internal reflections.

In order to increase futhermore the output brightness, we can add two mirrors in order to reduce the ouput face size as shown in the Fig. 3^25^. In this configuration, the light is also confined in the longitudinal direction leading then to a confinement in the three dimensions of space, hence the name "3D" configuration. By translating the mirrors in a plane parallel to the $w.t$ plane (Fig. 3), the output aperture surface, denoted $s$, can be varied from $s$ = $S$ = $w.t$ = 22 × 1 mm^2^ to $s$ = 0.55 × 1 mm^2^ corresponding to an aperture ratio $r=S/s$ varying from $r$ = 1 to $r$ = 40. These mirrors are beveled to avoid light aperturing in the case of very small apertures.

The next parts present results and analysis in the "2D" configuration (i.e. without mirrors on the output face) and in the "3D" configuration (i.e. with mirrors on the output face).

## Performance and analysis in the "2D" configuration

**Figure 4:**
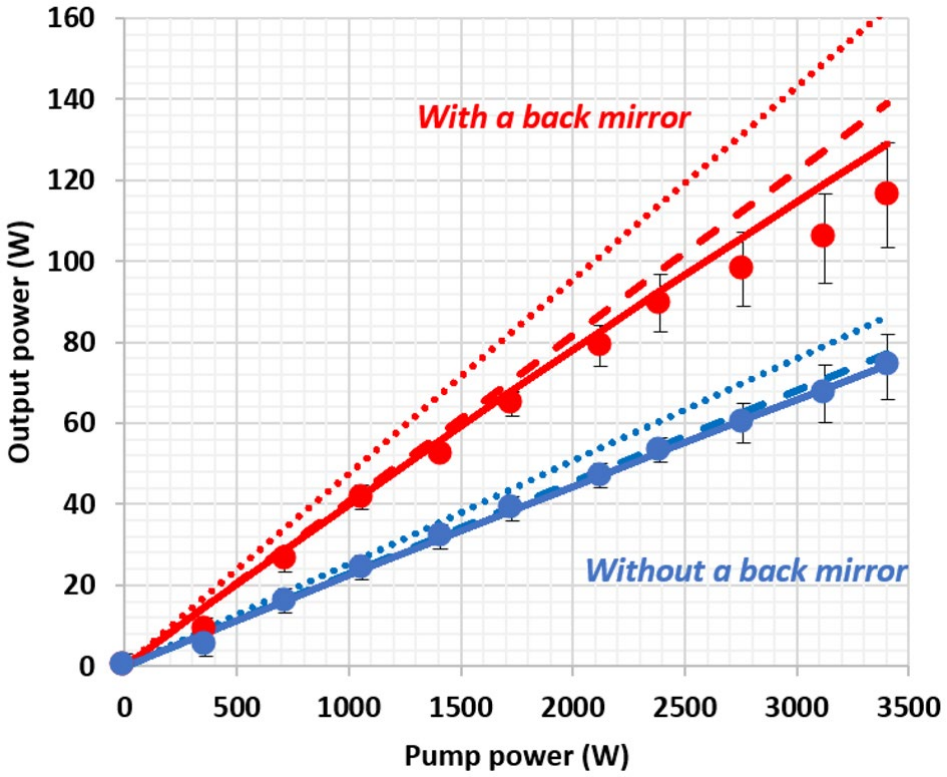
Peak power emitted by the LC versus the pump peak power with and without “back” mirror. Dots are experimental measurements. Plains curves are simulations including excited state absorption (ESA) and self-absorption losses. Dashed lines are simulations taking into account the self-absorption losses but not the ESA. Dot lines are simulations without taking into account ESA nor self-absorption.

The output peak power is measured with an integrating sphere coupled to a spectrometer (LCS-100 from Labsphere). As the output beam is Lambertian and strongly divergent, the entrance of the integrating sphere is positioned very close to the output surface for accurate measurements. The evolution of the output peak power versus the pump peak power is reported in Fig. 4, with and without the “back” mirror. The maximum peak power is 116 W for a pump peak power of 3405 W corresponding to a brightness of 167 W cm^–2^ sr^–1^ and an optical efficiency of 3.4%. In comparison, a Ce:YAG material in a similar setup provides an optical efficiency of 7.7%^12^.

In order to understand the origin of the limited performance, we modelized the output power as follows. Self-absorption losses are taken in account by separating the emission in two contributions P_1_ and P_2_, with respect to the propagation distance in the crystal.

P_1_ is the power related to "one-way" photons that are emitted in the output escape cone of the $w.t$ output face.

P_2_, is the power related to photons emitted in the backward emission cone and that propagate towards the output face after a reflection on the back mirror.

The expression of P_1_ is found by integration between z = 0 and z = *l* of all the emission in the output cone.

4 $$P_{1} = T_{LED} .\eta_{r} \frac{{\lambda_{p} }}{{\lambda_{em} }}P_{pump} { }\left( {1 - e^{{ - t\alpha_{p} }} } \right)\frac{{1 - cos\theta_{TIR} }}{2}\frac{1}{{\alpha_{1} Kl}}\left( {1 - e^{{ - \alpha_{1} Kl}} } \right)$$

where $${T}_{LED}$$ is the transmission of the $$w.l$$ face for the LED: As the LED emission cone is 30°, this quantity can be approximated by Fresnel transmission at normal incidence ($${T}_{LED}=0.91)$$, $${\lambda }_{p}$$ is the average pump wavelength (367 nm), $${\lambda }_{em}$$ is the average emitted wavelength (455 nm), $${P}_{pump}$$ is the peak pump power (from 0 to 3405 W), $${\alpha }_{1}$$ is the loss propagation coefficient for these photons propagating from 0 to $$l$$ in the concentrator. The loss has 3 contributions:

5 $${\alpha }_{1}={\alpha }_{0}+{\alpha }_{l}+{\alpha }_{ESA}$$

where α_0_ corresponds to the losses of the concentrator including the crystal quality, including diffusion loss coefficient and the polishing quality (α_0_ = 4 × 10^–3^ cm^−1^), ${\alpha }_{l}$ corresponds to the self-absorption losses $({\alpha }_{l}$ = 1.7 × 10^–2^ cm^-1^) and is calculated using spectral absorption data presented in Fig. 2, ${\alpha }_{ESA}$ corresponds to the losses induced by excited state absorption ${\alpha }_{ESA}={\sigma }_{ESA}{n}_{2}$, ${n}_{2}$ being the population density of the upper state level. As in^12^, we neglect the decrease of ${n}_{2}$, caused by ESA because of the low pump power density of LEDs. Therefore, the relation between ${n}_{2}$ and the pump power ${P}_{pump}$ is:

6 $${n}_{2}=\tau \frac{{P}_{pump}{T}_{LED}}{lwt}\frac{{\lambda }_{p}}{hc}\left.{\left(1-e\right.}^{-t{\alpha }_{p}}\right)$$

where τ is the lifetime of the upper level (τ = 41 ns).

The expression for P_2_ is similar to the expression for P_1_ but with an extra reflection on the opposite face and an additional propagation between *l* and *2l* in order to reach the output face.

In the case of a back mirror, the expression of P_2_ is:

7 $${P}_{2}={RP}_{1}{e}^{-{\alpha }_{2}Kl}$$

where

$${\alpha }_{2}={\alpha }_{0}+{\alpha }_{2l}+{\alpha }_{ESA}$$

${\alpha }_{2l}$ corresponds to the self-absorption losses for a propagation between *l* and *2l.* It is calculated similarly as ${\alpha }_{l}$ by considering the spectral density ${I}_{l}(\lambda )$ and ${I}_{2l}(\lambda )$ presented on Fig. 2$({\alpha }_{2l}$= 9 × 10^–3^ cm^−1^).

Without the back mirror, a Fresnel reflection on the opposite face must be considered, the expression of P_2_ is therefore:

8 $${P}_{2}={(1-{T}_{Fresnel})P}_{1}{e}^{-{\alpha }_{2}Kl}$$

where ${T}_{Fresnel}$ is the Fresnel transmission of the $w.t$ interfaces taking all directions in the output escape cone and all polarizations in account (${T}_{Fresnel}$ = 0.86).

For the calculation of the output power, we consider the light transmitted by the output $w.t$ interface (related to ${T}_{Fresnel}$) and the light reflected by this interface coming back after a reflection on the opposite face. Multiple reflection light can be simulated as in^25^. It corresponds to propagation lengths in the concentrator larger than $2l$.

Therefore, the output power with a back mirror having a reflectivity $R$ is:

9 $${P}_{2Dmirror}=({P}_{1}+{P}_{2})\frac{{T}_{Fresnel}}{1-{e}^{-2{\alpha }_{3}Kl}R\left(1-{T}_{Fresnel}\right)}$$

where ${\alpha }_{3}$ correspond to losses for photons propagating distances larger than $2l$ in the concentrator ${\alpha }_{3}={\alpha }_{0}+{\alpha }_{3l}+{\alpha }_{ESA}$

For those photons, self-absorption losses are estimated from our measurements presented in Fig. 2. Considering the spectral density ${I}_{2l}(\lambda )$ and ${I}_{3l}(\lambda )$ a loss coefficient namely ${\alpha }_{3l}$ can be deduced $({\alpha }_{3l}$= 4 × 10^–3^ cm^−1^). As the spectral shape evolves only slightly between $2l$ and $3l$, we can suppose that this value can also be used for longer propagation distances than $3l$.

Without the back mirror, the output power is found by replacing $R$ by $1-{T}_{Fresnel}$:

10 $${P}_{2D}=({P}_{1}+{P}_{2})\frac{{T}_{Fresnel}}{1-{e}^{-2{\alpha }_{3}Kl}{\left(1-{T}_{Fresnel}\right)}^{2}}$$

The simulations are presented in Fig. 4. In order to separate the different limitations, we calculate also the output power with and without excited-state absorption (ESA) and self-absorption. Figure 4 shows that self-absorption has a stronger influence than ESA. ESA effect is more visible with the back mirror since longer propagation distances render this configuration more sensitive to losses.

The simulations are adjusted to the experimental data thanks to three loss parameters that have not been measured: the effective reflectivity R of the back mirror (taking the large divergence of the output beam in account), the cross section of the excited-state absorption and the effective loss coefficient ${\alpha }_{0}$ taking in account crystal imperfections (diffusion measured to be 10^−3^ cm^−1^ and polishing quality). For a better accuracy, those parameters are adjusted by fitting the output power obtained in the "3D configuration" (see section “Performance and analysis in the 3D configuration”) which is much more sensitive to losses because of long propagation distances inside the concentrator. Despite the indirect adjustment, Fig. 4 shows that the 2D configuration simulations agree with experimental value $s.$

## Performance and analysis in the "3D" configuration

**Figure 5:**
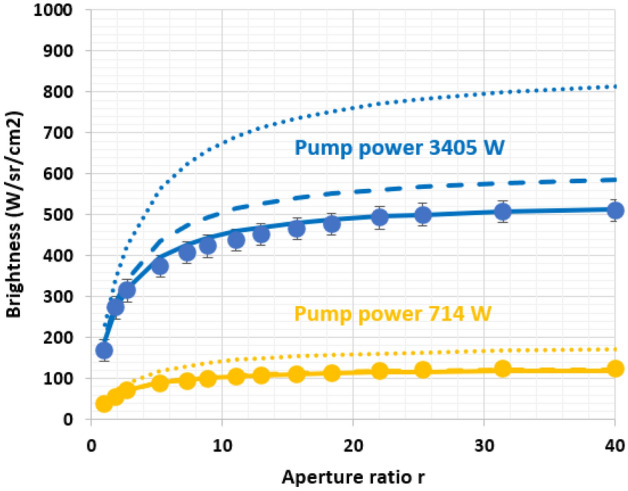
Brightness of the output surface for two different pump peak powers versus the aperture ratio. Dot are experimental values. Plains curves are simulations including excited state absorption (ESA) and self-absorption losses. Dashed curves are simulations without ESA but with self-absorption losses. Dot curves are simulations without ESA and without self-absorption.

In this section, we have measured the output power by translating the mirrors along the output face (see Fig. 2). At full aperture (aperture ratio r = 1) and full pump peak power (3405 W), we measure an output power of ${{P}_{3D r=1}=P}_{2Dmirror}$ = 116 W. At r = 22 corresponding to a squared output surface of 1 mm^2^, the output peak power decreases to ${P}_{3D r=22}$ = 16 W. This effect is mainly due to the propagation losses inside the Ce:LYSO. However, the brightness (W cm^–2^ sr^–1^) is increased by a factor 4. Light recycling in this particular "3D" configuration with r = 22 has two assets: first it increases the brightness, and second it symmetrizes the output surface in a square shape. Figure 5 gives the output brightness versus the aperture ratio for two different pump powers.

Assuming that the output emission is Lambertian, the brightness of the output area can be deduced:

11 $${B}_{3D}=\frac{{P}_{3D}.r}{\pi .w.t}$$

where ${P}_{3D}$ is the power emitted by the reduced output surface s and r is the aperture ratio r = S/s.

The output power ${P}_{3D}$ can be expressed by light recycling of P_1_ and P_2_ after every bounce on the mirrors (having the same reflectivity R)^25^:

12 $${P}_{3D}=\frac{1}{r}\left({P}_{1}+{P}_{2}\right)\frac{{T}_{Fresnel}}{1-{e}^{-2{\alpha }_{3D}d}\left[\frac{{1-T}_{Fresnel}}{r}R+\left(1-\frac{1}{r}\right){R}^{2}\right]}$$

where ${\alpha }_{3D}$ is the loss coefficient for propagation distances longer that 3 $l$, taking the light confinement in 3 dimensions in account. Compared to ${\alpha }_{3}$, self-absorption losses are adjusted at ${\alpha }_{inf}$= 3 × 10^–3^ cm^−1^. ${\alpha }_{3D}$ is therefore: ${\alpha }_{3D}={\alpha }_{0}+{\alpha }_{inf}+{\alpha }_{ESA}$.

The other fitting parameters are adjusted as follows.

R is adjusted for the low pump power (714 W) where the effect of ESA is the lowest. We found R = 92%. One must mention that this value is smaller than the mirror specifications (reflectivity of 99.3% at normal incidence). This smaller value can be related to an imperfect and partial reinjection of the recycled rays by the mirrors, due to the Lambertian emission of the concentrator.${\sigma }_{ESA}$ is adjusted for the high pump power (3405 W). We found ${\sigma }_{ESA}=2.5\times {10}^{-17} {\mathrm{cm}}^{2}$. One must note that this value is just an estimation and may include other effects depending on the pump power.

Once again, simulations are carried out under different conditions: with or without ESA and self-absorption. In this 3D configuration, ESA has a much greater impact than in 2D configuration. This can be explained by the long distance travelled by rays before reaching the output. However, self-absorption appears to be much more important limitation.

## Discussion

This work aims to better understand why a Ce:LYSO concentrator is less efficient than a Ce:YAG concentrator. Table 1 summarizes different parameters for these two crystals. Ce:LYSO has 3 parameters limiting the efficiency. By order of importance, the first one is the quantum efficiency, but this parameter could be improved by using Ce:LYSO crystals free of tetravalent cerium and specifically prepared to optimize quantum yield and not scintillation yield. The second one corresponds to self-absorption losses that limit the performance when the propagation distance increases, this parameter is intrinsic to the small Stokes shift of the Ce:LYSO in regard to the other garnet values (around 2600 cm^−1^ for Ce1 center and 3200 cm^−1^ respectively^26^) and is related to the blue emission of the Ce:LYSO in regard to the green/yellow in the case of Ce:YAG. The third one is the excited state absorption, limiting the performance when the pump power increases.

Despite these limitations, Ce:LYSO can be compared to blue LEDs as shown in the Table 2. For a fair comparison, LED performance are measured in the same temporal regime (40 µs 10 Hz) allowing to overdrive the LED and to obtain a peak power 2.6 times the specified power in CW. This value is limited by peak power saturation above a certain supply current.

**Table 2:** Comparison of performance of Ce:LYSO and a state-of-the-art blue LED (Rubix line by Lumiled).

Parameter description	Ce:LYSO	Blue LED
Dimensions (mm)	22 × 105 × 1	1 × 1
Pump pulse duration	40 µs	40 µs
Repetition rate	10 Hz	10 Hz
Output at full aperture ${P}_{2Dmirror}$	116 W	4.25 W
Brightness at full aperture	168 W cm^–2^ sr^–1^	135 W cm^–2^ sr^–1^
Output at squared 1mm^2^ aperture ${P}_{3D}$	16 W	4.25 W
Brightness at squared 1mm^2^ aperture	509 W cm^–2^ sr^–1^	135 W cm^–2^ sr^–1^

In the 2D configuration, Ce:LYSO concentrator offers the power of approximately 30 blue LEDs in a bright line of 1 × 22 mm^2^ with a brightness higher than the one of a single LED. One has to note that a LED system emitting the same power (116 W) would emit on a line of 1 mm × 60 mm assuming a state-of-the-art filling factor (50%) for the LEDs. This LED line would have a reduced brightness of 67.5 W cm^–2^ sr^–1^ caused by this filling factor. Therefore, with a brightness of 168 W cm^–2^ sr^–1^ and a power of 116 W, Ce:LYSO concentrator offers an unique combination clearly overcoming the performance of LED system.

In the 3D configuration, and for an aperture of 1 mm^2^, the Ce:LYSO concentrator delivers a power of 16 W with a brightness of 509 W cm^–2^ sr^–1^. To reach the same power with LEDs, a combination of 4 LEDs is then necessary. Assuming a filling factor of 50%, the square of 4 LED will measure 2.5 × 2.5 mm^2^ and will have a brightness of 80 W cm^–2^ sr^–1^, six times smaller than the brightness of the Ce:LYSO output surface.

**Figure 6:**
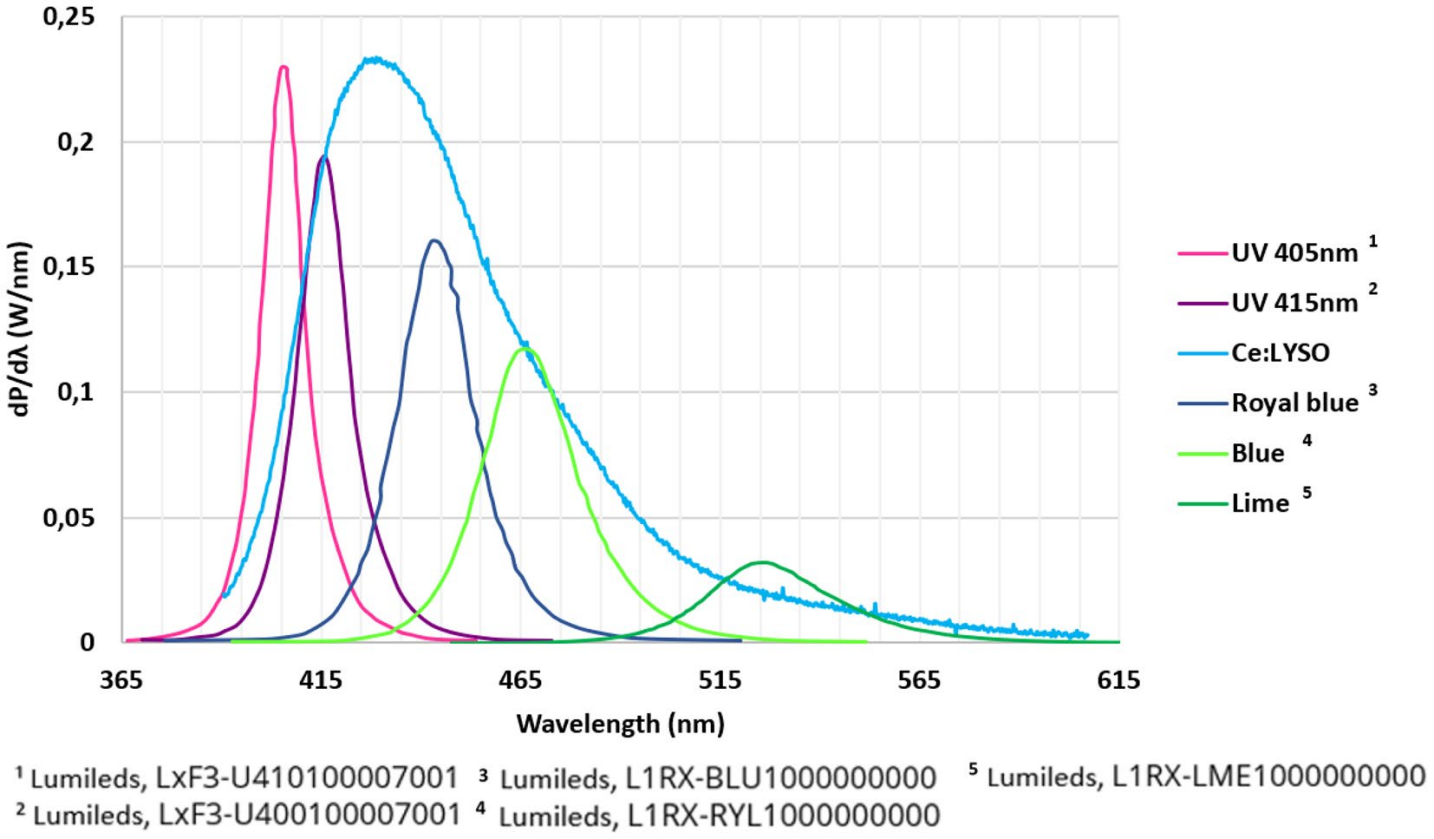
Spectral density of Ce:LYSO LC (square output surface of 1 mm^2^) and different LEDs.

Figure 6 compares the spectral power density of the Ce:LYSO luminescent concentrator (square output surface of 1 mm^2^) to the ones of several LEDs operating in the same QCW regime (i.e. peak power 2.6 times the specified power in CW). It shows that the Ce:LYSO luminescent concentrator covers the performance of 4 LEDs (typically from 415 to 530 nm) with similar spectral power density. In comparison, spectral combining of LEDs would certainly use more than 4 LEDs to smooth spectrum and would barely overcome the brightness of one single LED regarding the complexity to propagate a LED beam in an optical device (large size and large divergence).

In conclusion, this work explores the potential of the scintillator material Ce:LYSO as a LED luminescent concentrator for solid-state lighting. An approach combining experiments and numerical simulations allows to explain the performance of Ce:LYSO. Indeed, low quantum efficiency, strong self-absorption and excited-state absorption affect the output power and the brightness, that are 2–4 times smaller than for the well-known Ce:YAG luminescent concentrator for the same pump power and for similar dimensions. However, Ce:LYSO luminescent concentrator combines power and brightness at a level that is not reachable by any geometrical assembly of blue LEDs. Besides, Ce:LYSO performance could be improved by using single crystals specially prepared for optimized quantum yield and not for scintillation properties where the presence of Ce^4+^ is preferred. This work also gives some outlook on the search of other Ce^3+^ doped blue emitting light concentrators. Better host than Ce:LYSO will have to combine higher quantum yield and stokes shift typically greater than 3000 cm^−1^.

Anyway, this new powerful and bright source could address applications in the field of LED illumination. As LEDs, a Ce:LYSO concentrator is an incoherent source providing speckle noise free-illumination with a larger acceptability than laser sources regarding light safety limits. So, Ce:LYSO luminescent concentrator could be seen as an interesting way to "magnify" the blue LED in terms of brightness and spectrum.

With such a broad spectrum in the blue, Ce:LYSO emission overlaps the absorption spectra of many dyes used in standard fluorescence microscopy. Preliminary investigations of confocal microscopy are reported with LEDs of 1 W cm^–2^ sr^–1^ brightness^27^. As the Ce:LYSO luminescent concentrator offers a brightness 500 times higher, it has a much larger potential for this application.

The short lifetime of Ce in LYSO (41 ns) makes the source able to emit 100 ns pulses. It can be used in photo-acoustic imaging where the duration of the illumination pulse limits the spatial "in depth" resolution: 100 ns corresponding to a spatial resolution of 150 µm^28^. Indeed, LED photo-acoustic imaging has a strong potential especially in the blue for high contrast in-vivo mapping of vasculature networks in biological tissue^29^. However, the power density of LEDs limits the imaging depth (typically 1 cm). Ce:LYSO could therefore be a solution for exploring more clinical applications in the area of breast imaging and cardio-vascular medicine.

## Data Availability

Data underlying the results presented in this paper are not publicly available at this time but may be obtained from Lisa Lopez upon reasonable request.
